# Different Methods of Scan Alignment in Erosive Tooth Wear Measurements: An In Vitro Study

**DOI:** 10.3390/dj12020034

**Published:** 2024-02-05

**Authors:** Nikolaos Loumprinis, Stavroula Michou, Christos Rahiotis

**Affiliations:** 1 School of Dentistry, National and Kapodistrian University of Athens, 11527 Athens, Greece; 2 Department of Odontology, University of Copenhagen, 2200 Copenhagen, Denmark; stavroula.michou@sund.ku.dk

**Keywords:** scanner, wear, monitoring, alignment, mesh, energy drinks, best fit, reference best fit

## Abstract

Background: Model alignment in cases of erosive tooth wear can be challenging, and no method has been reported to outweigh the others. Methods: Extracted human teeth were mounted on two models and scanned at different times, from 1 h to 2 weeks, with an intraoral scanner (3Shape TRIOS 4) before and after immersion in Monster^®^ energy drink and tap water. The scans were superimposed (3Shape TRIOS Patient Monitoring, Version 2.2.3.3, 3Shape A/S, Copengagen, Denmark). Best fit, best-fit tooth comparison, reference best fit using fillings, and palatal rugae as reference points were used for alignment. Surface profile differences were calculated in a cross-section view. The nonparametric Bland–Altman and Kruskal–Wallis tests were used. Results: First, statistically significant differences were marked after 4 days of immersion. The measurements obtained after 2 weeks of immersion were statistically significantly different from the measurements obtained at the different time points until 1 week. No statistically significant differences (*p* < 0.05) were observed among the alignment methods at any time. Conclusion: In comparison to the best-fit model, both palatal rugae and fillings can be used. The best-fit tooth comparison method is a reliable option; however, it should be used with caution in cases of major surface loss.

## 1. Introduction

Quantitative analyses of outcomes and therapeutic concepts in dental disciplines are critical in evaluating treatment plans and possible failures. For these reasons, currently, the superimposition of digital dental scans is frequently employed [1]. Orthodontic tooth movement [2], the efficacy of aligner treatment [3], tooth wear monitoring [4], and even volumetric changes following soft tissue grafting [5] are only a few of the utilities digital technology provides us.

Erosion is one of the most typical causes of tooth wear, and it is defined as the dissolution of dental hard tissues caused by non-bacteriogenic acids [6]. When enamel undergoes an acid challenge, mineral loss starts in the carbonate-rich inter-prism space, creating surface softening, subsurface softening, and eventually an etching pattern. The latter leads to increased mechanical wear. Frequent exposure to these acids leads to progressive teeth softening and cumulatively irreversible tissue loss [7]. 

Tooth wear, especially erosive wear, is becoming an increasing problem these days as modern nutritional habits favor energy drink consumption [8]. Their highly erosive dynamic provokes tooth surface loss, often not detected in the early stages by clinicians. 

Energy drinks claim to boost concentration and physical performance and reduce sleepiness. These characteristics, in combination with a pleasant taste, make them quite popular among young people [9]. The caffeine and its extracts in these drinks reach up to five times more than a cup of coffee. They may contain the amino acid taurine, glucuronolactone, minerals, and glucose [10]. These drinks have an extrinsic acidity and a pH of up to 2.5 because of the quantity of carbonic acid formed by adding CO_2_, which produces fizz and other acids, such as citric acid, phosphoric acid, and tartaric acid. Therefore, the early detection and monitoring of tooth wear and quantifying the progression of tooth wear are of increasing importance in providing appropriate patient care.

The early signs of enamel erosion consist of a smooth, silky glazed surface without perikymata [11], which is a complex state to detect clinically and often evades detection. To overcome this problem, many methods have been introduced for the quantitative and qualitative assessment of tooth surface loss. These methods can be categorized as *in vitro*, *in vivo*, or a combination of both [12]. Digital profilometry is a reliable way of measuring wear *in vitro*, but the high cost and time-intensiveness inhibit its use in clinical practice [13]. Photographs and clinical indices are the main *in vivo* methods; however, they are considered semi-quantitative, as they do not measure exact tissue loss, and the examiner’s subjectivity influences the outcome [14]. In recent years, intraoral scanners (IOSs) have been used not only as restorative but also as diagnostic tools, with the help of special software. Model alignment/superimposition is a method for the qualitative and quantitative investigation of hard- and soft-tissue changes. 

In the past, most studies focusing on superimposition accuracy errors did not use a fully digital workflow. Gypsum models were scanned using a laboratory scanner [15,16,17,18]. More and more studies are using directly scanned models [4,19,20]. Regardless, the superimposition of two different datasets is prone to error. Also, not having a universal metric system makes studies even harder to compare. Efforts has been made in overcoming alignment errors; however, they still need further improvement. 

Three types of model alignment are used in dentistry: the best-fit alignment, the landmark alignment, and the reference best fit. The best-fit alignment, one of the first methods described [21,22], is based on an iterative closest point (ICP)-matching algorithm. In the ICP method, an iterative search of two datasets with the nearest points is performed. After that, the algorithm is used to estimate the rigid transformation, which aligns the models (global matching). Since the introduction of the ICP algorithm, many modifications and updates have been published [22], with each construction company and software using a slightly different algorithm. For example, 3Shape (3Shape A/S, Copenhagen, Denmark) has introduced an alignment method used for tooth comparison based on the best-fit alignment; however, this method only enables the recognition and alignment of the teeth, not the whole scanned model (local matching). The landmark alignment is widely used in the dental industry, especially when precision at the micrometer level is not essential. It is a straightforward procedure fundamentally based on the operator. However, when the points are not chosen correctly, the alignment will only partially be completed. Lastly, the reference best-fit method aligns datasets by allowing the operator to choose the points least likely to have changed [17,23]. It is a method that avoids alignment errors by minimizing the number of defects of interest to be measured but introduces operator bias when selecting the points of interest. According to a recent systematic review [24], this method prevails over the other two previously explained methods. Reference alignment leads to significantly lower alignment errors and more accurate measurements. By contrast, the best-fit and landmark alignment methods may underestimate the size of defects [25].

Parameters such as the matching method, the region of interest selection procedure, and the quality of the initial alignment impact the accuracy of the final alignment [23]. Registration techniques can be categorized into marker-based [23] and marker-free [26] approaches, with the first applied mainly in full-arch implant impressions. No marker can reliably be used in case of long-term wear monitoring, making scan registration quite challenging, especially when stable anatomical points are required. Teeth constitute the only hard tissue in the mouth. However, their use in the long-term monitoring of wear or orthodontic/restorative treatments may appear questionable [23]. 

Palatal rugae or plicae palatinae are irregular, asymmetric ridges of connective tissue located behind the incisive papilla [27]. They are considered the most stable anatomic part of the oral cavity, especially the third rugae. Minor changes may occur only with the skeletal expansion of the maxilla during orthodontic treatment [28]. Dental restorations also are considered as reference points for alignment. Composite resins are more durable against erosive and/or abrasive challenges than human enamel [29], and they have already been used as reference points in alignment protocols.

To our knowledge, no study directly compares different alignment methods with the commercially available IOS software TRIOS Patient Monitoring by 3Shape TRIOS A/S. This method can be used chairside in a clinical scenario. In the present study, the erosive wear process was assessed on semi-arch models of natural teeth. Different alignment points for the reference best fit, as well as global and local best-fit methods, were compared. The quantification of alignment error is yet to be performed, as well as the impact this error may subsequently have on metrics. The null hypothesis was that no statistically significant differences would be found between the alignment methods.

## 2. Materials and Methods

### 2.1. Specimen Preparation

Fourteen human teeth (*n* = 14) were used for this experiment. Dental students extracted all of them from Athens Dental School, National, and Kapodistrian University of Athens, as all had a poor prognosis due to terminal periodontitis. These teeth were collected as leftover biological material, and no notification to the National Ethical Committee was required. Teeth were stored in chloramine T trihydrate 0.5% *w*/*v* for a week until use.

A semi-arch model of the upper jaw (central incisor–second molar) was digitally modified to fit natural teeth. Two identical models were 3D-printed, and natural teeth were then mounted using acrylic resin-Jet^®^, Lang Dental Inc., Wheeling, IL, USA. Each semi-arch model resulted in having seven teeth, from the central incisor to the second molar. Composite resin restorations (Filtek Supreme XTE 3M ESPE, 3M Seefeld, Deutschland GmbH) were placed in three teeth of each model as follows: central incisor (IV class distally), canine (V class labially), and second molar (II class proximally). The casts were stored in a darkroom in a phosphate-buffered saline solution for hydration and under steady pH (Composition: NaCl 137 mmol/L, KCl 2.7 mmol/L, Na_2_HPO_4_ 10 mmol/L, and KH_2_PO_4_ 1.8 mmol/L; pH 7), at 37 °C until the initiation of the experiment.

### 2.2. Erosive Solutions and 3D Scanning 

Both semi-arch models were scanned with TRIOS 4 (3Shape TRIOS A/S, Denmark) to establish the baseline points. Afterward, they were immersed in one of two solutions: Monster^®^ (Monster Beverage, Corp., Corona, CA, USA) or tap water (control). Monster^®^, according to its manufacturer, contains the following ingredients: carbonated water, sucrose, glucose syrup, acid (citric acid), natural flavorings, taurine (0.4%), acidity regulator (sodium citrate), panax ginseng root extract (0.08%), l-carnitine l-tartrate (0.04%), caffeine (0.03%), preservatives (sorbic acid and benzoic acid), color (anthocyanins), vitamins (B2, B3, B6, and B12), sodium chloride, D-glucuronolactone, guarana seed extract (0.002%), inositol, and sweetener (sucralose). pH was measured at 3.48. For each designated time (1 h, 3 h, 6 h, 12 h, 1 d, 2 d, 4 d, 1 w, and 2 w), the models were removed from the solution, rinsed with deionized water for 1 min, dried with oil-free compressed air for 20 s, and scanned by the same operator to evaluate erosion. The models were then immersed again in the solutions. The times for solution immersion were strictly followed. In summary, each model was assessed at baseline and after time-controlled solution immersion. The experiment was performed in a room with a controlled temperature of 20 °C.

### 2.3. Alignment Methods

**Figure 1:**
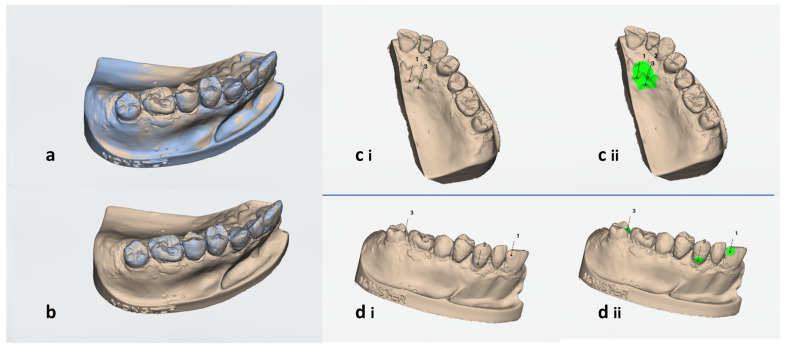
Different methods of alignment were used in the current study with Patient Monitoring Software: (**a**) best fit, (**b**) best-fit tooth comparison, (**c**) palatal rugae reference best fit, and (**d**) filling reference best fit. For reference best-fit alignment (**c**,**d**), first, three points were set (**ci**,**di**), and then the area was painted with a brush (**cii**,**dii**, green color).

The scanned models obtained at different times were superimposed with their baseline using Patient Monitoring Software (3Shape TRIOS^®^ Patient Monitoring, Version 2.2.3.3, 3Shape A/S, Copenhagen, Denmark). Best fit (global matching), best-fit tooth comparison (local matching), fillings, and palatal rugae (reference best fit) were used as the four alignment methods. For the first two methods, the operator did not decide on the best-fit alignment. In the third one, the operator manually marked the fillings by first marking points under the “three-point alignment” in TRIOS software and then marking the whole surface of the fillings using a brush. Similarly, for the fourth alignment method, the palatal rugae were marked (Figure 1).

Subsequently, each model was superimposed and compared with its baseline for all selected times (from 1 h to 2 weeks). A cross-section tool was used for two parts of each tooth—one mesially and one distally in a vertical direction. Four profile differences on the buccal surface and four on the palatal rugae were then calculated for each cross-section. In this way, sixteen measurements for each tooth, as small as 0.01 mm, were taken for the 3D models. Cross-sections were maintained for each tooth over the different time points to make the measurements more accurate. The procedure was repeated four times so that each alignment method’s measurements could be acquired.

### 2.4. Precision Assessment

Before the main experimental procedure, one of the semi-arch models was scanned from the same operator five times. These scans were superimposed on Patient Monitoring Software (3Shape TRIOS^®^ Patient Monitoring, Version 2.2.3.3, 3Shape A/S, Conpehagen, Denmark) using best fit, best-fit tooth comparison, fillings, and palatal rugae as reference points. As explained above, sixteen measurements of surface profile differences for each tooth were acquired. This method was selected to measure the precision of the final measurements.

### 2.5. Statistics

The Kolmogorov–Smirnov test was performed to examine if the data were normally distributed. As they did not follow a normal distribution, the nonparametric Kruskal–Wallis test was conducted to compare the surface profile differences among the different semi-arch models. Bonferroni correction was used to adjust significant values for multiple tests. The Bland–Altman analysis was also performed, which quantified the difference in agreement between measurements at 2-week intervals using a graphical method. 

The level of significance was set to a = 0.05. SPSS Statistics 26.0 (IBM Corp., Armonk, NY, USA) was used for statistical analysis. 

## 3. Results

The results for the different methods of alignment are shown in Table 1. First, statistically significant differences were recorded at 4 d for Monster^®^, and the measurements obtained at 2 w were statistically significantly different from those obtained at 4 d and 1 w. Regarding tap water, all measurements were 0.01 mm for surface loss, apart from the measurement of the 2 w time point for tooth comparison (0.02 mm), and no statistically significant difference was observed. No statistically significant differences were observed between the three alignment methods at any of the time points.

**Figure 2:**
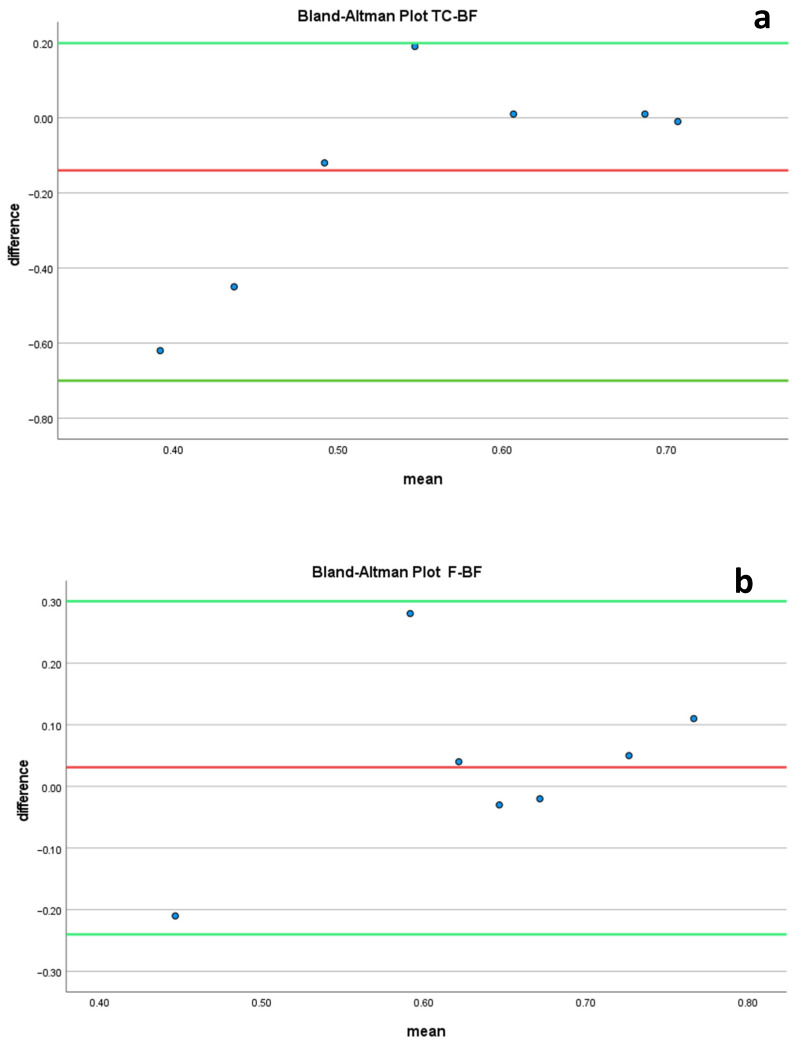

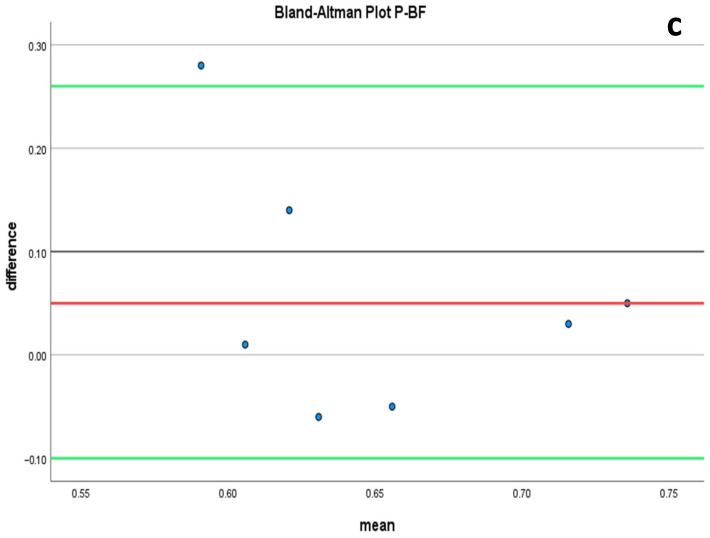
Bland–Altman plots comparing the differences between (**a**) reference best fit (BF)–tooth comparison (TC) scores with the average of TC-BF scores; (**b**) best fit (BF)–fillings (F) with the average of BF-F scores; and (**c**) reference best fit (BF)–palatal rugae (P) with the average of BF-P scores. *(Green lines: ±SD Confidence Interval lines, Red line: mean deference line)*.

The graphical plot of the Bland–Altman analysis is shown in Figure 2. 

The Bland–Altman analyses demonstrated excellent agreement between the reference best-fit method and the other alignment methods (*p* < 0.01).

**Figure 3:**
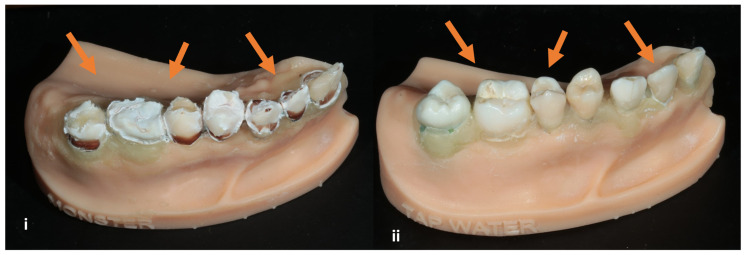
One-week tooth erosion through immersion using Monster^®^ energy drink (**i**), in contrast with teeth’s appearance after two-week immersion in tap water (**ii**). The arrows show the teeth’s appearance in both conditions.

After a 2-week immersion of a model, 0.62 mm, 0.45 mm, 0.67 mm, and 0.59 mm of surface loss was observed with best fit, best-fit tooth comparison, fillings, and palatal rugae reference best fit, respectively. The eroded teeth are visible in a visual examination, as shown in Figure 3.

**Figure 4:**
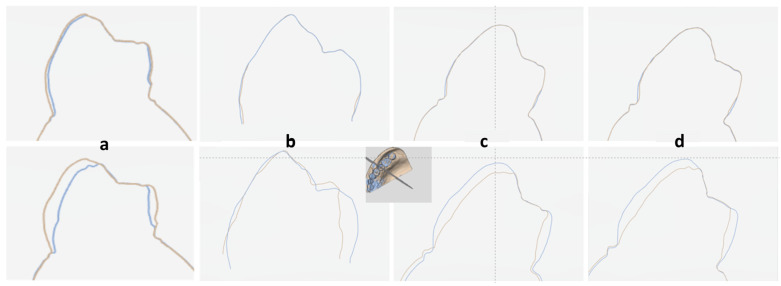
Cross-sections of the second premolar after immersion in Monster^®^ for 1 week (**upper**) and 2 weeks (**lower**): (**a**) best fit, (**b**) tooth comparison, (**c**) fillings, and (**d**) palatal rugae.

Cross-section views of the 3D models at different time points superimposed with the baseline are shown in Figure 4. In the 1-week energy drink immersion, differences were barely observed, and all alignment methods yielded accurate results. In the 2-week energy drink immersion, more distinct misalignment errors occurred, especially for the tooth comparison method.

## 4. Discussion

The present study aimed to assess different methods and points of scan alignment for erosive wear measurements. Based on the present results, no statistically significant differences were found; therefore, the null hypothesis is verified. 

In recent decades, growing evidence shows a considerable increase in the consumption of potentially erosive energy drinks [30]. The global estimation of erosion indicates that 30–50% of the population suffers from tooth erosion [31], and it is expected to increase due to increased human life expectancy, lifestyle changes, and people retaining their natural teeth for longer. Many observational studies found an association between energy and soft drink consumption and erosive wear severity [32,33,34]. Yet, only a few studies have investigated the quantification of tooth loss due to wear [14,35,36], and none of the studies has examined tooth loss due to energy drink consumption using intraoral scanners and semi-arch models. For tooth erosion to be reproduced and surface loss to be observed, in our study, an energy drink with high erosive potential [35] was chosen. At the 2-week time point, depending on the investigation method, surface profile loss from 0.45 mm to 0.67 mm was observed. 

Regarding precision, 3Shape manufacturers provide an uncertainty limit of around 50 μm. The larger the scan model is, the more significant the errors that are expected. In a study [37] with a similar protocol and semi-arch models, the precision measurement was calculated to be +/−10 μm. Additionally, our control group (tap water) measurements were around 0.01 μm, not zero. This is a number within the uncertainty threshold and agrees with the findings of another study [38].

Studies have shown relatively low progression in patients with physiological wear, with reported annual tissue loss of 11–29 μm [17,39]. In the case of pathological tooth wear, the surface loss was estimated between 68 and 140 μm per year [40], while patients suffering from erosion had a median of 36.5 μm over six months [41], all beyond the software’s uncertainty threshold.

No landmark alignment was investigated in the current study as this method had the lowest accuracy compared to the other two methods in terms of translation and angular errors. This also resulted in a statistically significant higher positive gain error [26]. 

The mean translation error resulting from the comparison between the original dataset and the realigned one was calculated to be 130 μm for the best-fit alignment and 22 μm for the reference best-fit alignment. The angular error was also estimated to be 0.56° for the best-fit alignment and 0.26° for the reference best-fit alignment [26]. Perfect realignment is challenging, and software and techniques are constantly developing.

In our study, two different sequences were used. Tooth comparison is an automatic selection method based on a local approach, namely tooth-to-tooth alignment where each tooth is used as a reference and a comparison is performed at the tooth level; thus, each tooth is aligned and compared separately, but all teeth are simultaneously shown on Patient Monitoring. The best-fit alignment and manual filling/palatal rugae selection methods are based on a global approach, with the best fit considered the reference standard for scan superimposition [21]. It involves jaw-to-jaw alignment and compares all scanned surfaces and teeth simultaneously. According to the literature [42], scanner inaccuracies significantly affect the methods using the global approach. Our results revealed no difference between the methods. Yet, fillings were distributed evenly, and palatal rugae comprised a relatively large part of the model, and this may be the reason why no statistically significant differences were observed between the two methods in the early stages. As erosive wear progressed with time, the opposite phenomenon was observed. The greater the surface defect was, the more inaccurate the best-fit alignment would be. As two scans of the same tooth are aligned at different time points, the software assumes that the geometries are equal. Thus, in case of significant defects, especially in the best-fit local approach option, the software may move the geometries to compensate for the deviation; therefore, an average superimposition occurs. In this way, even if a buccal “gain” is observed after an erosive challenge, a considerably more significant surface loss will be marked palatally. This is confirmed based on our results, as shown in Figure 4. No statistically significant difference in surface loss was found, which may be due to average superimposition. On the other hand, in cases of major surface losses, the best-fit global approach qualitatively yielded better results than the tooth comparison method, which is likely because a large part of the jaw incurs no damage from the energy drink, even though no statistically significant differences were observed. Another study [43] using the best-fit–tooth comparison option showed that the factor defect area was also significantly associated with inaccurate measurements.

No statistically significant differences were observed between the best fit, tooth comparison, palatal rugae, and fillings. Yet, the reported surface loss for Monster^®^ after 2 weeks for the best-fit method was 0.62 mm, whereas this value was 0.45 mm for tooth comparison, 0.67 mm for fillings, and 0.59 mm for palatal rugae. The tooth comparison model calculated smaller tooth loss than the other three methods (0.22 mm difference in measurements with fillings). As shown in Table 1, the interquartile range also increased in the tooth comparison model, which may be due to larger misalignment errors, as explained in the previous paragraph. Even though no statistically significant difference was observed, not all methods were found to be equally useful in a clinical scenario of wear monitoring with major surface loss.

In cases of long-time monitoring, a filling may undergo erosive/abrasive wear [44] and/or enzymatic catabolism [45]. The degradation of composite resins is in direct relation with the storage solution [46]. It is shown that the pH of the immersion solution can accelerate the erosive wear of polymers. Many factors can influence the behavior of restorative materials in acidic conditions [47,48]. Many studies have shown that Coca-Cola or Coca-Cola-like beverages are among the most aggressive erosive agents. Composite resin’s degradation in low pH solutions is due to hydrolytic degradation [49]. The first stage in erosive degradation is water absorption, which can further infiltrate the resin matrix at the interface with fillers or other defects, compromising their reinforcing effects. Also, factors like the cross-linked nature of the resin matrix and the solvent sorption uptake directly affect composite resin degradation [50]. Previous studies showed that the increase in composite resin roughness in acidic solutions is probably due to their softening surface, which leads to the leaching of the resin components and the displacement of the filler particles [51]. It has been confirmed that, due to erosion, the softened zone of the tooth’s enamel is quite susceptible to mechanical forces, which otherwise only have a minor effect on the native enamel’s surface. Previous studies also showed a direct correlation between the size of the filler and the amount of material loss [52]. The shape of the fillers can also influence the resistance of the composite resin to abrasive challenges. In our study, we used Filtek Supreme XTE) with a matrix composed of bis-GMA, UDMA, TEGDMA, and bis-EMA(6) resins. Zirconia fillers of 4 to 11 nm and silica fillers of 20 nm were incorporated.

In terms of palatal rugae as a reference point, on the other hand, its prominent structure makes it clinically detectable. Its mobility level is relatively low due to its high collagen content. However, small changes may be observed, primarily in cases of age growth and orthodontic treatments involving maxilla expansion. On the other hand, there are no data regarding moisture and dimensional change after pressure. Our protocol involved the use of 3D-printed palatal rugae, which may have some limitations in clinical practice. Attention must be paid to choosing the proper reference points to reach clinically accurate conclusions.

Ideally, the registration technique must be non-invasive, not time-consuming, automatic, and straightforward. In recent years, significant advances have been made in this field. Yet, perfect alignment is difficult to obtain, as no perfect point of alignment has been introduced in cases of long-term monitoring. This study has some limitations. First, we only evaluated the performance of TRIOS 4 and the accompanying TRIOS Patient Monitoring Software. Other software packages may result in better or worse alignment accuracy. To date, only a few studies have compared different software for alignment [53,54], and none included 3Shape TRIOS Patient Monitoring. Second, only two semi-arch models were investigated; a future study with a larger sample would be of interest. We also used semi-arch models, not full-arch ones; in a clinical scenario of wear, a full-arch scan is undoubtedly essential. Using a full-arch scan allows for a more balanced distribution of reference points; however, more significant inaccuracies are expected as the scanning area is doubled. 

Progressive tooth wear with severe exposure to dentine is a restorative drawback for patients, especially for young patients who want to maintain as much tooth structure as possible. Changes in patients’ lifestyles and the restorative processes needed are challenging for dental practitioners, as a holistic rehabilitation program is unquestionable [55]. Early diagnosis is of utmost importance, as it gives dentists the opportunity to implement only non-operative preventive programs. Diagnostic windows for detecting qualitative change in study models, clinical indices, clinical photographs, and intraoral scans range from 18 months to 2 years [56]. Commercial tooth wear analysis software will likely override the need to record a clinical wear index if you take intraoral scans. However, documenting a clinical index is prudent until then or if working with an analog workflow. Different software programs exist for the quantitative measurement of tooth wear, with intraoral scans potentially diagnosing active wear in six months. However, current problems with scan registration accuracy and measurement limit their diagnostic potential. 

## 5. Conclusions

The surface loss of semi-arch models was measured with intraoral scan software. Different methods and reference points were used, and according to our results, no statistically significant differences were found. Still, in cases of large defects, choosing the most suitable scan alignment method and measurement metric is of high importance for proper diagnosis and monitoring. 

## Figures and Tables

**Table 1:** Median surface profile differences (mm) and interquartile range (IQR) at different times (h: hour, d: day, and w: week), after immersion in Monster^®^ and tap water. Letters ^a,b,c^ show the statistically significant differences between the groups at different time points.

Time	Monster^®^				Tap Water			
	Best Fit	Tooth Comparison	Fillings	Palatal Rugae	Best Fit	Tooth Comparison	Fillings	Palatal Rugae
1 h	0.01 (0) ^a^	0.01 (0) ^a^	0.01 (0) ^a^	0.01 (0) ^a^	0.01 (0) ^a^	0.01 (0) ^a^	0.01 (0) ^a^	0.01 (0) ^a^
3 h	0.01 (0) ^a^	0.01 (0) ^a^	0.01 (0) ^a^	0.01 (0) ^a^	0.01 (0) ^a^	0.01 (0) ^a^	0.01 (0) ^a^	0.01 (0) ^a^
6 h	0.01 (0) ^a^	0.01 (0) ^a^	0.01 (0) ^a^	0.01 (0) ^a^	0.01 (0) ^a^	0.01 (0)	0.01 (0) ^a^	0.01 (0) ^a^
12 h	0.01 (0) ^a^	0.01 (0)	0.01 (0)	0.01 (0) ^a^	0.01 (0) ^a^	0.01 (0) ^a^	0.01 (0) ^a^	0.01 (0) ^a^
1 d	0.01 (0) ^a^	0.01 (0) ^a^	0.01 (0.01) ^a^	0.01 (0) ^a^	0.01 (0) ^a^	0.01 (0) ^a^	0.01 (0) ^a^	0.01 (0) ^a^
2 d	0.05 (0.02) ^a^	0.02 (0.05) ^a^	0.02 (0.03) ^a^	0.01 (0.01) ^a^	0.01 (0) ^a^	0.01 (0) ^a^	0.01 (0) ^a^	0.01 (0) ^a^
4 d	0.13 (0.04) ^b^	0.08 (0.07) ^b^	0.1 (0.14) ^b^	0.08 (0.12) ^b^	0.01 (0) ^a^	0.01 (0.01) ^a^	0.01 (0) ^a^	0.01 (0) ^a^
1 w	0.27 (0.07) ^b^	0.22 (0.07) ^b^	0.24 (0.08) ^b^	0.21 (0.1) ^b^	0.01 (0.05) ^a^	0.01 (0) ^a^	0.01 (0) ^a^	0.01 (0) ^a^
2 w	0.62 (0.09) ^c^	0.45 (0.2) ^c^	0.67 (0.11) ^c^	0.59 (0.07) ^c^	0.01 (0.03) ^a^	0.02 (0.02) ^a^	0.01 (0) ^a^	0.01 (0) ^a^

## Data Availability

The data presented in this study are available on request from the corresponding author.
